# Exploring Influence Of Window Views, Nature And Context On Hong Kong’s Care Home Residents’ Psychosocial Well-being

**DOI:** 10.1093/geroni/igaf122.3284

**Published:** 2025-12-31

**Authors:** Mohana Das, Peter Hasdell

**Affiliations:** The Hong Kong Polytechnic University, Hong Kong, China (People’s Republic); The Hong Kong Polytechnic University, Hong Kong, China (People’s Republic)

## Abstract

This study investigates the psychosocial impact of window views, nature access, and culturally mediated spatial dynamics on older adults’ Quality of Life (QOL) in Hong Kong’s (HK) hyperdense Long-term Care Facilities (LTCFs). The study evaluates how environmental factors affect residents’ sense of belonging, wellbeing, and disengagement in care homes with spatial constraints and window access restrictions. The COVID-19 pandemic’s impact on confined spaces underscored the importance of indoor environments to mitigate social isolation. A methodologically rigorous mixed-methods approach integrates cross-sectional surveys (n = 408) assessing nature and view perceptions, semi-structured interviews (n = 33) examining lived experiences, and architectural audits of 6 LTCFs to evaluate space configurations. Although quantitative data supports Western literature on nature’s healing properties, qualitative and contextual analyses show that HK’s hyper-urbanized milieu, with inadequate green/blue infrastructure and people’s cultural acclimation to density, favoured socially dynamic urban panoramas over passive nature scenes. Architectural audits revealed systemic design flaws such as tokenistic nature integrations. The study proposes a socio-ecological gerontological design framework that challenges universalist beliefs about nature’s importance in well-being and emphasizes culturally appropriate models for hyperdense Asian cities. It presents context-specific design paradigms that prioritize spatial efficiency, dynamic urban imagery, and micro-nature integrations for high-rise limits by combining architectural analysis with psychosocial insights. These findings foster evidence-based LTCF initiatives and advocate for localized design standards and retrofitting solutions that address HK’s socio-spatial constraints. The findings indicate that well-being frameworks tailored to the specific contexts of non-Western, densely populated urban areas should be developed to effectively support their rapidly aging populations.

